# FTSNet: Fundus Tumor Segmentation Network on Multiple Scales Guided by Classification Results and Prompts

**DOI:** 10.3390/bioengineering11090950

**Published:** 2024-09-22

**Authors:** Shurui Bai, Zhuo Deng, Jingyan Yang, Zheng Gong, Weihao Gao, Lei Shao, Fang Li, Wenbin Wei, Lan Ma

**Affiliations:** 1 Institute of Biopharmaceutical and Health Engineering, Tsinghua Shenzhen International Graduate School, Tsinghua University, Shenzhen 518055, China; bsr22@mails.tsinghua.edu.cn (S.B.); dengz24@mails.tsinghua.edu.cn (Z.D.); hudenjear@gmail.com (Z.G.); gwh20@mails.tsinghua.edu.cn (W.G.); lifangmable616@163.com (F.L.); 2 Beijing Tongren Hospital, Capital Medical University, Beijing 100730, China; ccmu_yangjingyan@163.com (J.Y.); qlhp@hotmail.com (L.S.); weiwenbintr@163.com (W.W.)

**Keywords:** fundus tumor, segmentation, deep learning

## Abstract

The segmentation of fundus tumors is critical for ophthalmic diagnosis and treatment, yet it presents unique challenges due to the variability in lesion size and shape. Our study introduces Fundus Tumor Segmentation Network (FTSNet), a novel segmentation network designed to address these challenges by leveraging classification results and prompt learning. Our key innovation is the multiscale feature extractor and the dynamic prompt head. Multiscale feature extractors are proficient in eliciting a spectrum of feature information from the original image across disparate scales. This proficiency is fundamental for deciphering the subtle details and patterns embedded in the image at multiple levels of granularity. Meanwhile, a dynamic prompt head is engineered to engender bespoke segmentation heads for each image, customizing the segmentation process to align with the distinctive attributes of the image under consideration. We also present the Fundus Tumor Segmentation (FTS) dataset, comprising 254 pairs of fundus images with tumor lesions and reference segmentations. Experiments demonstrate FTSNet’s superior performance over existing methods, achieving a mean Intersection over Union (mIoU) of 0.8254 and mean Dice (mDice) of 0.9042. The results highlight the potential of our approach in advancing the accuracy and efficiency of fundus tumor segmentation.

## 1. Introduction

Over the past few decades, significant advancements in retinal imaging technology have greatly enriched our understanding of various retinal pathologies. Fundus tumors, as the most severe form of retinopathy, pose a high risk of blindness and can be induced by the metastasis of other cancers. Fundus tumors are a group of very serious diseases, with uveal melanoma being the most common type [1,2]. If not treated promptly, fundus tumors have a high potential to cause blindness. In the United States, the incidence rate of uveal melanoma is 4.3 per million people [3]. According to ten years of clinical statistics from The Eye Hospital of Wenzhou Medical University [4], tumor cases account for about 1.5% of all cases, and the proportion of people has been relatively stable over the decade. In addition to Optical Coherence Tomography (OCT) and Magnetic Resonance Imaging (MRI), fundus images can also be used to detect and diagnose retinal pathologies, including fundus tumors [5,6]. Compared with OCT and MRI detection methods, fundus images have the advantages of being easily obtained and cost-effective. Fundus tumor lesions usually appear in the peripheral area of the retina and exhibit significant vascular changes that can be identified in fundus images. Accurate measurement of tumor size and depth is crucial for diagnosis, a task traditionally performed by ophthalmologists, which is both time-consuming and labor-intensive. Therefore, automatic segmentation of fundus tumors has always been a hot topic of research. Computer-aided diagnostic tools can help simplify this process by automatically locating and segmenting tumors.

However, currently, there is no publicly available dataset specifically containing fundus tumor images, so we collaborated with Beijing Tongren Hospital to collect a new fundus tumor segmentation dataset named FTS, which includes 254 pairs of fundus images with benign or malignant tumor lesions and their reference segmentation images. Detailed information about the dataset can be found in Table 1.

**Figure 1:**
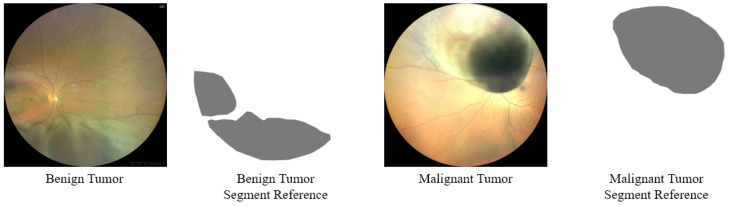
Samples of the Fundus Tumor Segmentation (FTS) image dataset.

**Figure 2:**
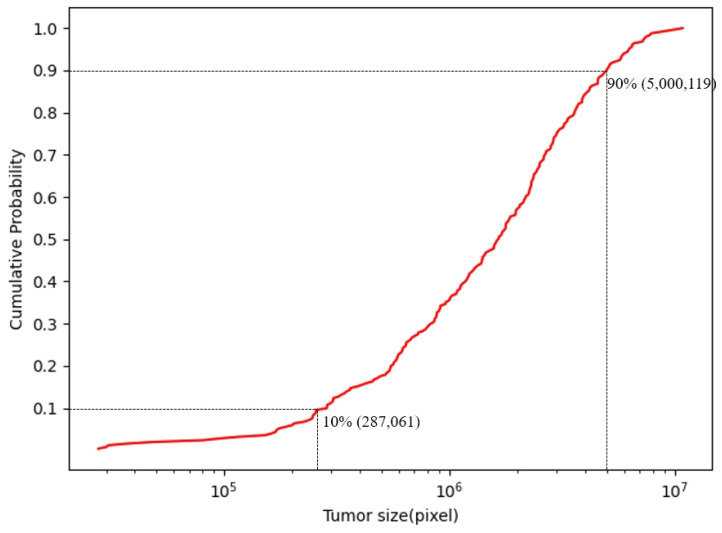
The cumulative distribution function of lesion sizes on the FTS dataset, which shows that the scale variation across them is enormous.

Moreover, compared with other types of medical image lesions, fundus tumors have the characteristics of varying lesion sizes and irregular lesion edges. As can be seen from Figure 1, the edges of the tumor lesions are irregular, and the overall shape of the lesions is also irregular. Figure 2 shows the cumulative distribution function of the size of tumor lesions in the FTS dataset, from which it can be found that the size of the largest and smallest tumors in the entire dataset can differ by more than a hundred times, and most of the lesion sizes are distributed in the range of 287,061 pixels to 5,000,119 pixels, which also differs by about 12 times. Consequently, the segmentation of fundus tumors is a very challenging task.

Therefore, there is an urgent need to develop a high-performance segmentation method tailored to the characteristics of fundus tumor imaging. To address the challenges posed by the significant changes in the size and shape of tumor lesions, we designed a segmentation network (FTSNet), which extracts features on multiple scales based on convolution and attention mechanisms. The core of our method is the design of an encoder capable of extracting image feature information on multiple scales, which combines convolutional operations with attention mechanisms to extract features from input images of different scales. To integrate information across various scales, we have designed a feature fusion module capable of merging features at different levels, particularly incorporating small-scale details into the global features extracted by the attention mechanism, which means that we can ultimately obtain a feature map that contains rich information at various scales. This fusion strategy ensures that the generated feature maps contain both global and local information, which is crucial for accurate segmentation. In addition, we also use the principle of prompted learning, adopting the text encoder of the Contrastive Language–Image Pretraining (CLIP) model [7], to formulate a dynamic prompt head for segmentation. This head is driven by prompt sentences and feature maps, and can adapt to the specific segmentation requirements of different lesion types and fundus images. The prompt sentence comes from a classification task performed on the extracted feature maps after feature extraction, combining the results with predefined prompt templates to form the final prompt sentence. In this way, we can achieve more refined and targeted segmentation results.

Our code is available at https://github.com/aqaaqaaqa/FTSNet.

## 2. Related Work

### 2.1. Deep Learning Segmentation Network

The emergence of deep learning has revolutionized the field of medical image segmentation, with neural networks serving as robust tools that enable end-to-end solution methodologies. A plethora of researchers have harnessed the capabilities of deep learning to innovate in the domain of medical image segmentation. Notably, the U-net architecture [8] has established itself as a paragon of excellence, prompting the development of several specialized derivatives. These include Unet++ [9], which enhances the original U-net framework; Attention U-net [10], integrating attention mechanisms to focus on relevant features; ResUNet [11], incorporating residual connections to facilitate deeper networks; and nnu-net [12], which further refines the segmentation process. Each of these variations contributes to the advancement of deep learning techniques in the precise delineation of pathological regions within medical images. For deep learning on ophthalmic medical imaging, Rformer [13] proposed a fundus image restoration network based on the attention mechanism, which exemplifies the advantages of the attention mechanism for fundus image information extraction. DCEN method [14] uses two different networks for classification and rating work on cataract fundus images and fuses the feature maps of these two networks, reflecting the importance of feature fusion.

In the field of segmentation, previous studies have shown excellent results on various medical images [15,16,17]. However, these methods can only be adapted after being retrained with fundus tumor data, limiting their widespread clinical application. In the field of fundus tumor segmentation, researchers have also paid attention to the use of OCT images, although these images have diagnostic value, they are costly to obtain [18]. Many community hospitals do not have the equipment to perform the OCT test, and patients have to travel to larger hospitals for treatment. Other studies have attempted to apply existing segmentation methods to fundus images to improve their performance on such images [19,20]. FNeXter [21] proposes a network that fuses the attention mechanism and convolution operations for lesion segmentation in OCT images, demonstrating that the fusion of these two mechanisms helps to improve model performance.

There is a paucity of research specifically dedicated to the segmentation of fundus tumors. Among the limited work in this area, Rahdar and colleagues have introduced a model based on semisupervised machine learning for the segmentation of retinoblastoma tumors, achieving satisfactory outcomes on their proprietary dataset. Their approach commenced with the utilization of a Gaussian Mixture Model to identify anomalies within a corpus of roughly 4200 ocular fundus images. The results garnered from this methodology were then employed to train an economical model aimed at fulfilling the same objective [20].

### 2.2. Prompt Learning in Computer Vision

Prompt learning has risen as a potent strategy for amplifying the efficacy of vast pretrained Natural Language Processing (NLP) models. Recognizing its profound capacity, investigators are progressively keen on examining the deployment of learnable prompts within VLMs (vision-language models). CLIP, as the pioneering large-scale pretrained model for image–text alignment, was nurtured on an impressive corpus of four billion image–text pairs [7].

Despite the plethora of initiatives in the computer vision arena that draw inspiration from the CLIP model, endeavors in the medical image segmentation sector have been comparatively sparse. ConVIRT [22] proposed an alternative unsupervised strategy to learn medical visual representations by exploiting naturally occurring paired descriptive text. The new method of pretraining medical image encoders with the paired text data via a bidirectional contrastive objective between the two modalities is domain–agnostic, and requires no additional expert input. Gloria [23] extracts features through the image and text encoders, and learns global and localized representations by contrasting attention-weighted image subregions and words in the reports. The learned global–local representations are utilized to obtain label-efficient models for various downstream tasks including image-text retrieval, classification and segmentation. Qin et al. have presented an exhaustive investigation into the transferability of knowledge from pretrained VLMs to medical applications [24], illustrating that adeptly crafted medical prompts are instrumental in effectively unlocking the knowledge embedded within pre-trained VLMs. Constructing upon this premise, J Liu et al. have advanced the CLIP-Driven Universal Model, which assimilates text embeddings derived from CLIP into segmentation models. This methodology is designed to confront the obstacles engendered by the diminutive scale and fragmented labeling of discrete datasets [25].

## 3. Method and Dataset

### 3.1. Dataset

This research collated the Fundus Tumor Segmentation (FTS) dataset with ocular fundus images obtained from the Ophthalmology Department of Beijing Tongren Hospital. The image size in the FTS dataset is 4304 × 4306. The fundus images are captured by ophthalmologists using a ZEISS VISUCAM200 fundus camera (ZEISS, Jena, Germany), which is a mainstream product of the fundus camera. For each image, two doctors will label the image separately. After the labeling is completed, the image and the results are handed over to an experienced doctor who chooses one of the labels and makes the final modifications to ensure the accuracy of the image labeling. The three doctors and the images are all from Beijing Tongren Hospital.

The FTS dataset includes two classification label categories: Benign Tumor and Malignant Tumor. The number of images in each category is presented in Table 1. A total of 254 images are categorized under Benign and Malignant Tumors, with the fundus images within these groups having been segmented by ophthalmologists for further refinement. This study was performed in line with the principles of the Declaration of Helsinki. The Ethics Committee of Beijing Tongren Hospital, Capital Medical University granted approval and supervised this research.

### 3.2. FTSNet

In this section, we elaborate on the operational dynamics of the model we propose. We commence with an overview of the model’s general architecture. We then proceed to expound upon the individual critical components of the model, encompassing the encoder, decoder, feature fusion module, and the prompt dynamic head.

#### 3.2.1. Overview

In the field of traditional neural networks, the distinct receptive fields of each layer facilitate the capture of information at multiple scales. However, deeper networks are vulnerable to the loss of small-scale information during the propagation process due to a series of computational operations. This challenge has been a key focus in the literature on object detection, prompting the evolution of architectural advancements like the Feature Pyramid Network (FPN) [26]. To address this issue, we have developed a multiscale feature extraction network.

**Figure 3:**
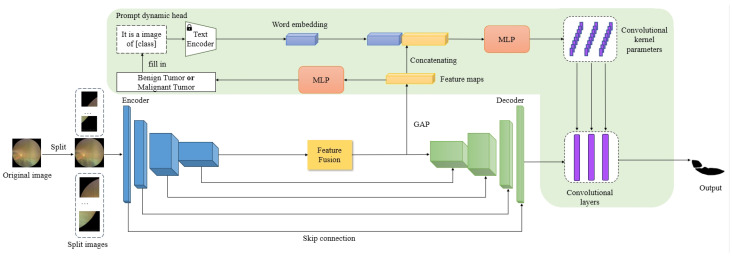
An overview of Fundus Tumor Segmentation Network (FTSNet). FTSNet adopts a U-shaped structure, composed of an encoder and a decoder. Both the encoder and decoder consist of four stages. The text encoder from the pretrained CLIP model, which is not modified during training. The Prompt dynamic head is an innovative component in our network that concatenates word embeddings with feature maps to dynamically generate the convolutional kernel parameters and bias parameters at the final convolutional layer. The feature maps are fed into an MLP to obtain a binary classification result, i.e., whether this image has a benign or malignant tumor, followed by filling the result into the prompt template.

The architecture of the entire model is depicted in Figure 3. The model employs an encoder-decoder framework, akin to the U-net architecture [8], which is renowned for its efficiency in image segmentation tasks. The model accepts an input image $I\in R^{3\times H\times W}$, where the dimensions represent the number of channels and the spatial extent (height and width) of the image, respectively.

Initially, the model segments the input image into three different scales: $I_{1}\in R^{3\times H\times W}$, $I_{2}\in R^{48\times \frac{H}{4}\times \frac{W}{4}}$, and $I_{3}\in R^{192\times \frac{H}{8}\times \frac{W}{8}}$. To meet the input specifications of the model, the channel dimension of image $I_{2}$ is adjusted by replication to align with the model’s required channel count, resulting in the final size of $I_{2}\in R^{96\times \frac{H}{4}\times \frac{W}{4}}$.

The rationale behind the chosen segmentation dimensions is guided by the size distribution of lesions as shown in Figure 2. The majority of lesions vary in size from 287,061 to 5,000,119 pixels. Consequently, the segmentation dimensions are carefully selected to be within a moderate range that can comfortably encompass the majority of lesions. This approach ensures that each lesion’s information is retained intact within a single image segment, which is conducive to a more thorough and precise analysis by the model.

By preserving an optimal segmentation size, the model is better positioned to learn from the complete context of each lesion, a factor critical for accurate feature extraction and segmentation outcomes. These segmented images are then fed into the encoder, yielding three feature maps $F_{1},F_{2},F_{3}\in R^{768\times \frac{H}{32}\times \frac{W}{32}}$ of uniform size. These feature maps are subsequently processed through the feature fusion module, culminating in the generation of fused feature maps $F\in R^{768\times \frac{H}{32}\times \frac{W}{32}}$.

This fused feature map *F* is then introduced into the decoder, yielding a feature map $F^{’}\in R^{3\times H\times W}$ that encapsulates a wealth of information from the image. Ultimately, a dynamic head pro-dyn-head, in conjunction with prompts, is applied to produce the final segmentation results, as defined by the equation:

(1) $$mask=pro\text{-}dyn\text{-}head(F^{’},Prompt,F).$$

This design is particularly beneficial for the segmentation of fundus tumor lesions, which are known for their considerable variability in size and shape. The model’s ability to handle such heterogeneity is a testament to its robustness and adaptability in medical image segmentation applications.

#### 3.2.2. Encoder

**Figure 4:**
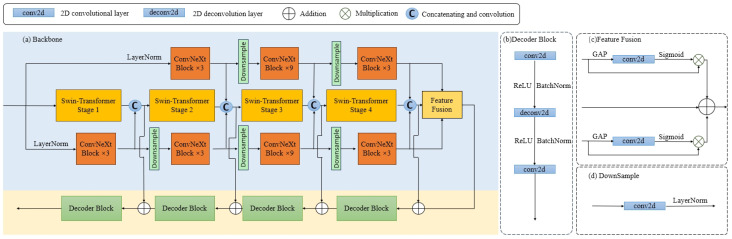
Detailed Backbone Network structure: (**a**) Backbone. This contains the encoder module, decoder module, and the feature fusion module. (**b**) Decoder Block. This component primarily comprises 1 × 1 convolutions, 3 × 3 transposed convolutions, and sequential 1 × 1 convolutions. (**c**) Feature Fusion. Feature fusion input in top-to-bottom order is the same as the output in encoder top-to-bottom order (**d**) DownSample. The downsampling operation is mainly performed by the convolution. Specific parameter settings are the same as ConvNeXt.

The encoder module is primarily designed to extract feature information from the image. The encoder comprises three main components, tailored to address the challenge of the vastly varying shapes and sizes of tumor lesions in fundus images. To enhance the model’s capability to extract features of varying sizes from fundus images, we have engineered an encoder that handles feature extraction for three distinct image sizes. For an in-depth look at the specific structure of the encoder, one can refer to Figure 4a.

The encoder’s cornerstone is a Swin-Transformer network [27] $Enc_{1}$, which takes in images of size $I_{1}\in R^{3\times H\times W}$. The primary goal is to harness the attention mechanism to extract global information from the entire image. The Swin-Transformer network [27] has been selected as the bedrock of our architecture due to its remarkable ability to extract global features via the attention mechanism. In contrast to traditional convolutional operations, which might be constrained in capturing long-range dependencies, the attention mechanism in the Swin-Transformer network excels at focusing on prominent features across the entire image. This ability to represent features holistically is essential for our encoder module, which aims to distill comprehensive global information from the input images. By utilizing the Swin-Transformer network’s hierarchical and shiftable attention mechanism, our architecture is adept at capturing the intricate spatial relationships inherent in the image data, thereby enhancing the overall feature extraction process.

Given the structural similarities between the ConvNeXt network [28] and the Swin-Transformer network, we have chosen a segment of the ConvNeXt network to train on $I_{2}$ and $I_{3}$, to learn feature information of the medium and small regions within the fundus image. This choice also capitalizes on the strengths of convolutional operations in capturing fine-grained features and the robust feature extraction capabilities of the ConvNeXt network. Image $I_{2}$ is fed into a module $Enc_{2}$, which is primarily based on the ConvNeXt architecture, except that the downsampling layer in *stage1* of the ConvNeXt is replaced with a module comprising a convolutional layer with a 1 × 1 kernel size and a layer normalization. Concurrently, $I_{3}$ is input into a structure $Enc_{3}$ that omits the *stage1* part of the ConvNeXt network and substitutes the downsampled layer in *stage2* with a structure that also includes a convolutional layer with a 1 × 1 kernel size and a layer normalization. This modification is primarily to accommodate the size of the input images $I_{2}$ and $I_{3}$. Such a network design also leverages the advantages of convolutional operations in extracting small features and the powerful feature extraction capabilities of the ConvNeXt network. The encoder ultimately produces three feature maps of the same size:

(2) $$F_{1}=Enc_{1}\left(I_{1}\right),\;F_{2}=Enc_{2}\left(I_{2}\right),\;F_{3}=Enc_{3}\left(I_{3}\right).$$

The specific structural parameters within our network are configured to match those of the ConvNeXtV2 Tiny and Swin-Transformer Tiny architectures. This approach ensures that our model leverages the proven effectiveness and efficiency of these well-established networks.

Our network’s encoder segments the original image into parts of different dimensions, which are then processed by various layers of the network. This method enables the precise extraction of information from localized regions within the image.

#### 3.2.3. Feature Fusion

To fully harness the information contained within the feature maps obtained by the network, the three feature maps $F_{1},F_{2},F_{3}$ derived from the network encoder undergo further feature fusion. This fusion process aims to reinforce the information about smaller areas within the fundus image present in $F_{2},F_{3}$, thereby enhancing the information in $F_{1}$, improving the model’s feature extraction capabilities, and aiding in subsequent segmentation tasks. To achieve this, we devised a feature fusion method that optimally utilizes the feature information in $F_{2},F_{3}$.

The feature map $F_{2}$ first undergoes a Global Average Pooling (GAP) operation to yield a result of size $R^{768\times 1\times 1}$. This result is then subjected to a 1×1 convolution operation conv followed by a Sigmoid function, and finally, a dot product operation ⊗ with $F_{2}$ to obtain the final result:

(3) $${\bar{F}}_{2}=\mathrm{sigmoid}\left(conv\left(\mathrm{GAP}\left(F_{2}\right)\right)\right)⊗F_{2}.$$

Similarly, for $F_{3}$, we perform the equivalent operations to achieve:

(4) $${\bar{F}}_{3}=\mathrm{sigmoid}\left(conv\left(\mathrm{GAP}\left(F_{3}\right)\right)\right)⊗F_{3}.$$

The final step involves adding ${\bar{F}}_{2}$ and ${\bar{F}}_{3}$ to $F_{1}$ to obtain the resultant feature map:

(5) $$F=F_{1}+{\bar{F}}_{2}+{\bar{F}}_{3}.$$

The architecture of our feature fusion module is significantly inspired by the Squeeze-and-Excitation Network (SENet) [29]. The module initiates with a GAP that condenses the spatial dimensions H×W of the input feature maps, creating a channel descriptor. This condensation encapsulates the information within the channel descriptor, reducing spatial redundancy while retaining essential feature information.

Post the GAP, a 1×1 convolutional layer coupled with a Sigmoid function is employed to modulate interlayer weight relationships, allowing the model to adaptively emphasize salient features. This process is particularly effective in enhancing the prominence of features at smaller scales, which is crucial for our focus on segmented image information.

The final additive operation integrates the processed information, facilitating a seamless fusion of multiscale features. This comprehensive fusion strategy ensures that the model adeptly captures the nuanced details of the image while maintaining its overall contextual integrity, thus bolstering the feature representation for subsequent tasks such as segmentation.

The feature fusion module accentuates the incorporation of small-scale details into the global context through an attention mechanism, assisting the network in concentrating on the most critical areas of the image for segmentation accuracy. Features extracted at different scales possess unique characteristics; small-scale feature maps may contain more intricate information about lesion edges, while large-scale feature maps provide information about overall shape and location. The fusion capitalizes on the strengths of these varying representations, ensuring that the final feature map *F* encompasses a rich array of information from both global and local perspectives, providing a solid foundation for the ensuing segmentation tasks.

#### 3.2.4. Decoder

The decoder segment adopts a more straightforward design compared with the encoder, primarily serving to reinstate the intricate semantic features extracted by the encoder. The skip connection mechanism effectively channels intricate details from the encoder to the decoder, mitigating the loss of information resulting from consecutive pooling and strided convolution operations. Akin to the U-net architecture, we integrate an efficient module to enhance the decoding capabilities. In U-shaped networks, two prevalent decoding techniques are simple upsampling and deconvolution. Upsampling enlarges the image dimensions through linear interpolation, whereas deconvolution (alternately known as transposed convolution) achieves this by executing a convolution operation. Notably, transposed convolution excels at learning adaptive mappings, enabling it to reconstruct features with superior detail. Consequently, we opt for transposed convolution within the decoder to restore features with heightened resolution. As illustrated in Figure 4b, this component primarily comprises 1×1 convolutions, 3×3 transposed convolutions, and sequential 1×1 convolutions. The channel configuration across the layers of our network is meticulously designed to correspond directly with the encoder’s specifications, following a sequence of [384, 192, 96, 3].

In our network, skip connections are implemented by directly summing the feature maps from corresponding parts of the encoder. The encoder’s feature maps are obtained by concatenating feature maps from three different branches, which capture information at various scales and levels of abstraction. However, after concatenation, the channel size of the resulting feature maps may change, necessitating a convolutional layer to adjust the dimensions back to their original size. This process is crucial for maintaining consistency in the spatial dimensions of the feature maps throughout the network, which is essential for subsequent operations such as decoding or segmentation tasks.

Leveraging the skip connection and the decoder module, the feature decoder ultimately generates a mask that aligns precisely with the dimensions of the original input $F^{\prime }$.

#### 3.2.5. Prompt Dynamic Head

The resulting output by the decoder module is considered a feature map enriched with various information about the image. In order to accomplish the final segmentation, a segmentation head is required to compute and segment the decoder’s result. We use a prompt dynamic head that is driven by both the prompts and the features *F* derived by the encoder. The prompts come from the combination of classification results and the prompt sentences.

A prompt is a sentence composed of English words: it is a image of *lesion class*. For our current task, there are two types of lesions, namely benign and malignant tumors. The classification of the tumors is derived from a classification network, which processes the feature maps output by the feature fusion module through a multilayer perceptron, ultimately obtaining the classification result for the image. Subsequently, the classification result is filled into the prompt template to obtain the final prompt sentence. The prompt is encoded through the text encoder of the CLIP model to obtain a word embedding vector prompt. The feature map *F* is first subjected to a GAP operation and then concatenated with the vector prompt to obtain a new vector proF. The new vector proF is computed through a module Cont consisting of three linear layers to obtain the convolution kernel parameters and bias parameters of the three convolution layers in the final segmentation head. So the final prediction of the model is

(6) $$\mathrm{result}=\mathrm{Prompt}\;\mathrm{dynamic}\;\mathrm{head}(F^{\prime },\mathrm{proF},\mathrm{prompt}).$$

In our methodology, the segmentation process is tailored to the specific characteristics of each lesion by assigning a unique set of convolutional kernel parameters to each type of pathology. This customized strategy is essential for achieving a high level of segmentation accuracy, as it enables the model to discern and delineate each lesion type based on its individual morphological and textural attributes. The use of lesion-specific convolutional kernels thus serves as a key differentiator in our approach, enhancing the model’s ability to accurately segment and classify diverse types of lesions in medical imaging.

## 4. Experiments

### 4.1. Experiment Setup

#### 4.1.1. Loss Function

In the training process, we employ a weighted sum of three loss functions as the final loss function. The final loss function is defined as

(7) $$Loss=\lambda_{1}L_{ce}+\lambda_{2}L_{dice}+\lambda_{2}L_{boundary\text{\_}dice}$$

Here, $L_{ce}$ represents the cross-entropy loss, which serves to measure the closeness between the model’s predicted probability distribution and the true distribution. It is defined as

(8) $$L_{ce}\left(y,p\right)=-\sum_{i}y_{i}\log\left(p_{i}\right)$$

Here, $L_{dice}$ represents the Dice loss function, which serves to measure the overlap between the predicted segmentation results and ground truth and is particularly useful for handling imbalanced segmentation data. It is expressed as:

(9) $$L_{dice}\left(y,p\right)=1-\frac{2\sum_{i}y_{i}p_{i}}{\sum_{i}y_{i}+\sum_{i}p_{i}}$$

where $y_{i}$ represents the true labels, and $p_{i}$ represents the predicted probabilities. The term *i* denotes the *i*-th pixel.

The Boundary Dice Loss is specifically designed to calculate the segmentation progress of the lesion boundary. Unlike traditional Dice Loss, Boundary Dice Loss involves dilating the labels and prediction results. The dilated results are then subtracted from the original to obtain the boundary area of the lesion. This boundary area is subsequently used as the input for the Dice function calculation. The main distinction from the Dice function is that it solely computes the boundary region.

(10) $$y_{i}^{\prime }=y_{i}-dilate\left(y_{i}\right)$$

(11) $$p_{i}^{\prime }=p_{i}-dilate\left(p_{i}\right)$$

(12) $$L_{boundary\text{\_}dice}\left(y^{\prime },p^{\prime }\right)=1-\frac{2\sum_{i}y_{i}^{\prime }p_{i}^{\prime }}{\sum_{i}y_{i}^{\prime }+\sum_{i}p_{i}^{\prime }}$$

where dilate means dilating operation. This approach emphasizes the importance of accurately delineating the edges of lesions, which is crucial for many medical image analysis tasks. By focusing on the boundary region, the Boundary Dice Loss can provide a more precise measure of segmentation performance in scenarios where the lesion’s edge is particularly significant for diagnosis and treatment.

#### 4.1.2. Evaluation Metrics

The FTSNet is a segmentation model, hence we employ Intersection-over-Union (IoU) in Equation (13) and Dice in Equation (14) to measure its performance on the FTS dataset. The metrics are averaged in the experiments; therefore, these are mean IoU (mIoU) and mean Dice (mDice) results in the experiments section.

(13) $$\mathrm{IoU}=\frac{\left|X\cap Y\right|}{\left|X\cup Y\right|}$$

(14) $$\mathrm{Dice}=\frac{2\times |X\cap Y|}{\left|X|+|Y\right|}$$

#### 4.1.3. Training Details

The original resolution of images is 4304 × 4306, which would be too large for our training purposes. Therefore, we initially resize the images and their corresponding labels to 448 × 448. Following the resizing, we randomly adjust the input images and their labels for hue, saturation, and brightness. Subsequently, we perform random transformations on the input images and their labels, including translation, zoom, rotation, and aspect ratio alteration. Our data augmentation strategy also incorporates random operations such as horizontal flip, vertical flip, and 90-degree rotation.

The initial learning rate was set to 0.0002 with a batch size of 12. The learning rate is halved if the model has been trained for 20 epochs and the loss remains nondecreasing. Training is terminated if the learning rate reaches $5\times 10^{-7}$ or if the model has been trained for 50 consecutive epochs without a reduction in the learning rate. We employed AdamW as the optimizer.

All experiments were conducted on four Nvidia RTX3090 GPUs (Nvidia, Santa Clara, CA, USA) with 24 GB of memory each. The Python version is 3.8.0, the PyTorch version is 2.0.0, and the CUDA version is 11.8.

### 4.2. Results

The models evaluated include FCN [30], U-net [8], Unet++ [9], ResUNet [11], DeepLab v3+ [16], TransUNet [17], Swin-Unet [31], and FTSNet, and specific results can be found in Table 2. Each model’s performance is quantified by its respective mIoU and mDice scores, which are crucial indicators of segmentation accuracy. The results demonstrate a range of performance levels, with FTSNet achieving the highest scores of 0.8254 in mIoU and 0.9042 in mDice, suggesting its superior performance in the task of image segmentation. FTSNet shows an improvement of approximately 1.49% over Swin-Unet in terms of mIoU and about 1.90% in terms of mDice.

In comparison with models that have similar means, our model has a smaller standard deviation, which indicates that our model has stronger robustness.

**Figure 5:**
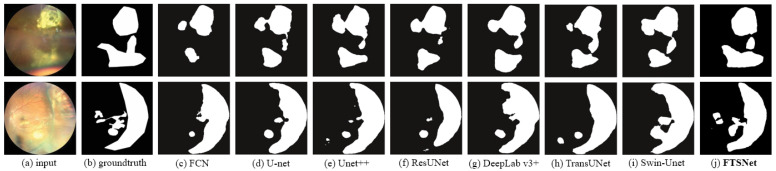
Comparison of segment results from different networks.

To offer an intuitive qualitative comparison, Figure 5 presents the segmentation outcomes of eight varied methodologies. A discernible observation from the illustration is that our FSTNet excels over alternative networks in capturing the morphology of tumor lesions and the minute details at the lesion boundaries, thereby showcasing the merits of our architectural approach.

### 4.3. Ablation Study

In the ablation study, we explored the effects of different loss function combinations and different encoder designs on the model results. The training details are the same as those written above, except for the parts we explicitly point out.

#### 4.3.1. Combinations of Loss Functions

As illustrated in Table 3, each constituent of the loss function imparts a positive influence on the model’s results. Notably, the Dice loss function has a more pronounced effect on the model than the boundary Dice loss function, underscoring the significance of the Dice loss function in the context of medical image segmentation tasks. Nevertheless, the boundary Dice loss function also provides a modest improvement in the model’s segmentation precision, highlighting its impact on the model’s ability to discern edges.

#### 4.3.2. Combinations of Encoder

As demonstrated in Table 4, the interchangeability between the Swin-Transformer network and the ConvNeXt network exerts little influence on the outcomes of the model. Utilizing the Swin-Transformer as the core network within the encoder yields a modest enhancement in performance relative to ConvNeXt, which reflects the role of the attention mechanism for global information extraction. Nevertheless, should only one of these networks be considered for the encoding process, there is a more distinct degradation in results, in which the Swin-Transformer is even more inferior compared with employing ResNet50 as the sole encoder. This observation may be attributed to the diminutive size of the FTS dataset.

## 5. Conclusions

This study introduces FTSNet, a pioneering segmentation network specifically tailored for the intricate task of segmenting fundus tumors in ophthalmology. The development of FTSNet marks a significant advancement in the field, offering a robust solution to the challenges posed by the diverse morphological characteristics of fundus tumors. Our approach, grounded in the principles of multiscale feature extraction and prompt learning, has demonstrated a remarkable capacity to accurately delineate tumor lesions, a critical factor in the diagnostic process.

Our experimental results are highly encouraging, with FTSNet outperforming several state-of-the-art models in terms of mIoU and mDice scores. The ablation studies further validate the effectiveness of our approach, particularly the impact of the loss function combinations and the encoder design on the model’s overall performance.

In conclusion, FTSNet represents a significant stride in the automation and enhancement of fundus tumor segmentation. Its ability to handle the variability and complexity of tumor lesions with high accuracy has the potential to greatly assist clinicians in diagnostic and therapeutic decision making. While our model has demonstrated impressive results, there remains scope for further optimization and expansion to other medical imaging domains. Future work will focus on refining the model’s generalizability, exploring additional pretrained models for prompt learning, and potentially integrating more advanced data augmentation techniques to bolster the model’s robustness.

## Figures and Tables

**Table 1:** Number of fundus images in each category on FTS.

Label	Benign Tumor	Malignant Tumor
Number	103	151
Percentage	40.55%	59.45%

**Table 2:** Quantitative comparisons with other methods. The format is metrics ± standard deviation.

Network	mIoU	mDice
FCN [30]	0.7587 ± 0.1067	0.8320 ± 0.0931
U-net [8]	0.7958 ± 0.1023	0.8721 ± 0.1128
Unet++ [9]	0.7905 ± 0.1385	0.8643 ± 0.1088
ResUNet [11]	0.7844 ± 0.1327	0.8599 ± 0.1490
DeepLab v3+ [16]	0.8077 ± 0.1426	0.8711 ± 0.1525
TransUNet [17]	0.8023 ± 0.1592	0.8731 ± 0.1451
Swin-Unet [31]	0.8133 ± 0.1804	0.8873 ± 0.1389
FTSNet	0.8254 ± 0.1266	0.9042 ± 0.1371

**Table 3:** Impact of different combinations of loss function.

Method	mIoU	mDice
only $L_{ce}$	0.7741	0.8452
$\lambda_{1}L_{ce}+\lambda_{2}L_{dice}$	0.8196	0.9017
$\lambda_{1}L_{ce}+\lambda_{2}L_{boundary\text{\_}dice}$	0.7859	0.8603
$\lambda_{1}L_{ce}+\lambda_{2}L_{dice}+\lambda_{2}L_{boundary\text{\_}dice}$	0.8254	0.9042

**Table 4:** The effect of different types of encoder networks on the results. The red tick indicates the network at the center of the encoder.

Swin-Transformer	ConvNeXt	ResNet50	mIou	mDice
		🗸	0.7965	0.8676
	🗸		0.8007	0.8723
🗸			0.7811	0.8490
🗸	🗸		0.8193	0.8919
🗸	🗸		0.8254	0.9042

## Data Availability

The FTS datasets generated and analyzed during the current study are not publicly available due to the Beijing Tongren Hospital protocol but are available from the corresponding author upon reasonable request.
